# Economic evaluation of the direct cost resulting from childhood poisoning in Morocco: micro-costing analysis

**DOI:** 10.1186/s13690-020-00440-z

**Published:** 2020-06-19

**Authors:** Fatima Zohra Benabdellah, Abdelmajid Soulaymani, Abdelrhani Mokhtari, Rachida Soulaymani-Bencheikh, Abderrazzak Khadmaoui, Hinde Hami

**Affiliations:** 1 Laboratory of Genetics and Biometry, Faculty of Science, Ibn Tofail University, Kenitra, Morocco; 2 Moroccan Poison Control Center, Rabat, Morocco; 3 Faculty of Medicine and Pharmacy, Mohammed V University, Rabat, Morocco

**Keywords:** Direct costs, Poisoning, Childhood poisoning costs, Morocco

## Abstract

**Background:**

The analysis of the economic burden for childhood poisoning has great value in Morocco where there still exists a paucity of information on the subject. The objective of this study was to explore the economic costs of unintentional and intentional poisoning in children in the region of Rabat-Salé-Kénitra, Morocco.

**Methods:**

A prospective study of children younger than 15 years with a poisoning diagnosis conducted between March and July 2016 in the Children’s University Hospital of Rabat, Morocco. The source of data for this study was questionnaire that collected information on the costs, the epidemiological and the socio-economic characteristics of childhood poisoning.

**Results:**

Eighty-seven patients were interviewed during the study period (39 females and 48 males). The majority of poisoning cases (98.85%) were accidental and 1.15% were intentional. Drugs, snake bites and scorpion stings, carbon monoxide, food, plants, household products, illegal drugs, pesticides, petroleum and industrial chemicals products were caused the poisoning. Of all the hospitalized patients, 77 (88.5%) were admitted to the emergency department and 5 (5.7%) were admitted to the intensive care unit. The average direct medical costs and the average direct non-medical costs of providing poison treatment were USD 127 and USD 30 per child, respectively. Total average direct cost of USD 157 (127 + 30) represented 60% of the national minimum wage per month in Morocco. Total direct medical costs accounted for 80%, as against 20% direct non-medical costs. The mean ± SD length of stay (LOS) for children with poisoning was 2.15 ± 1.87 days with a range variated between 0 day and 10 days.

**Conclusions:**

Overall, this study confirms that the costs of childhood poisoning are not negligible costs in Morocco. Therefore, the prevalence and the costs of childhood poisoning can be reduced by monitoring an open communication between parents, the Poison Control Centre of Morocco (MPCC) and physicians in order to increase the vigilance of parents against the risks related to unintentional poisoning that can be prevented with more awareness.

## Background

Poisoning has an important impact on health and well-being of children. It is associated with high rates of disability, death and significant economic costs [1, 2]. In 2017, the Poison Control Centre of Morocco (MPCC) reported 44,850 additional cases of poisoning [3] including snakebites and scorpion stings which represented 62% (27,944 cases due to snakebites and scorpion stings) of all cases. This institute revealed that the proportion of children aged less than 15 years represented 26.5% (11,885 cases) of all cases.

Health is a major element of human capital that impacts the economic outcomes. According to Baker and Stabile (2011), every child has a specific stock of health which decreases by diseases, injuries, mental and physical conditions [4], while parental investments in children made in terms of money and time help in sustaining the children’ stock of health. In the context of poisoning, the environment in which the child lives (urban or rural), the parents’ illiteracy, the parents’ wealth and resources, the parents’ access to information and technologies and so on are factors that significantly impact the prevalence and burden of childhood poisoning worldwide at various rates from a country to other [5].

The hypothesis assumes that the unintentional and intentional poisonings are related to significant social and economic burdens. Following the Institute for Health Metrics and Evaluation (IHME) contribution to the worldwide burden of diseases, the Global Burden of Disease study (GBD) has shown an overall number of 180,000 deaths in 2017 due to the unintentional poisoning [6]. SmartRisk (2009) found that the economic burden of unintentional childhood poisonings in Canada was approximately USD 771 million from which USD 281 million was attributed to direct costs [7]. Durkin et al. (1994) confirmed that childhood poisonings are related to significant economic burden, in fact, the indirect costs due to poisoning in children under 15 years in Northern Manhattan was almost USD 400 million, with an average direct and indirect costs of USD 1780 for each child [8]. Other studies conducted in South Africa estimated the average direct costs of poisoning to USD 106.50 per patient in urban Pretoria [9] and USD 75.58 in the Cape Peninsula with a total direct costs of children’s hospitalization because of paraffin poisoning to USD 1.4 million per year [10]. While those international studies have provided a deep cost-analysis and an average cost of poisoning burden, such studies were not undertaken before in Morocco and are, consequently, of significant value.

This study explores the direct costs of poisoning among children from patient perspective in the Rabat-Salé-Kénitra region, Morocco. It attempts to describe the epidemiological characters and the costs of childhood as well as to compare these costs according to its different components and the type of hospital admissions.

## Methodology

This is a hospital-based prospective study, the interview took place in the Children’s University Hospital of Rabat, Morocco. In total, one hundred cases with poisoning diagnosis were reached the hospital from March to July 2016 whether hospitalized or not, from which eighty-seven accepted to participate in the study. The face-to-face interview with parents was used to obtain data in order to lead quantitative analysis that describes the epidemiological characters of childhood poisoning as well as direct medical costs (examinations, physician visits, length of stay, medication costs) and direct non-medical costs (transportation, food and companion’s/caregiver’s hotel stays). The Children’s University Hospital of Rabat is the only hospital for children with a regional vocation.

### Subjects

During the study period, eighty-seven children younger than 15 years, with poisoning diagnosis attended the hospital were eligible for inclusion in the study. Children were excluded from the study if the parental consent was not obtained. A written consent for participation was individually distributed to parents before the process of data collection in order to ensure confidentiality and anonymity of participants.

### Ethics

The ethical standards of the study were approved by the Ethics Committees for Biomedical Research (CERB), Faculty of Medicine and Pharmacy of Rabat; clearance number-IORG0006594. Informed consent was obtained from all participants. The respondents provided signed consent form before their participation to the survey. Each potential respondent was properly informed of the questionnaire objectives, the related aspects to the study and the research procedure. The follow-up survey was carried out during the entire length of hospital stay.

### Data collection

The collection of data started from the child’s arrival to their departure from the hospital. In the current study, the children interval of age variate between [0–15[ instead of [0–18[ since during the study period no adolescent with poisoning diagnosis was received to the hospital.

The questionnaire aimed to underline the importance of poisoning burden in Morocco. The first section collected the sociodemographic characteristics of patients and their companions (dates of birth, sex, region and city of provenance and parent’s employment). The next section of the questionnaire concerned the clinical data filled out every day during the length of hospital stay. The final section of the questionnaire explored the direct costs incurred during the length of hospital stay. Indirect costs are out of the scope of this study.

The average medical cost per poisoning child was calculated based on all the expenditures incurred including (physicians visits, laboratory and imagining tests, cost of hospital stays, drug prescriptions). The average non-medical cost per poisoning child was calculated based on all the expenditures incurred including (transportation costs to and from the hospital, food costs and companion’s hotel stays) [11]. Moreover, the sum of direct medical costs and direct non-medical costs for all poisoning cases was calculated. All costs were indexed to 2016 United States Dollars (USD).

Costs presented in tables as means with standard deviations (SD). All data were manually recorded before being registered and analysed using the IBM SPSS Statistics version 25.

## Results

### General characteristics

A total of eighty-seven poisoning children attended the hospital during the period of the study (55% males and 45% females). Children less than five years presented 67% of poisoning cases with a sex ratio (M/F) of 1.2. A proportion of 71% of cases occurred in urban areas against 29% in rural areas and they were mainly from the Rabat-Salé-Kénitra region. In this study, patients were mainly of low socio-economic status with a percentage of 64.4% against 32.2% of middle socio-economic status.

Table 1 shows that childhood poisoning occurred mainly via oral exposures with 65.5% followed by dermal exposures with 24,1% then inhalation in 10.3% of the patients. Moreover, drugs, snake bites and scorpion stings, carbon monoxide and household products were the main causes of childhood poisoning that represented respectively 21.8, 24.1,10.3 and 10.3%. The intentional poisoning presented 1.15% of poisoning cases.

**Table 1:** Childhood poisoning characteristics (*N*=87) at the Children’s University Hospital of Rabat between March and July 2016

Patients characteristics	Number of cases	Frequency (%)
**Gender**		
Male	48	55.2
Female	39	44.8
**Age**		
< 1 year	9	10.3
[1–5 years[	49	56.3
[5–10 years[	14	16.1
[10–15 years[	15	17.2
**Residence location**		
Urban	62	71.3
Rural	25	28.7
**Region of provenance**		
Tanger-Tétouan-Al Hoceima	2	2.3
Rabat-Salé-Kénitra	77	88.5
Casablanca-Settat	8	9.2
**Economic status**		
Lower	56	64.4
Middle	28	32.2
Upper	3	3.4
**Routes of exposure**		
Oral exposure	57	65.5
Inhalation	9	10.3
Dermal exposure	21	24.1
**Poisoning agents**		
Drug	19	21.8
Snake bite and scorpion stings	21	24.1
Carbon monoxide	9	10.3
Food	5	5.7
Plant	3	3.4
Household product	9	10.3
Illegal drug	3	3.4
Pesticide	7	8.0
Petroleum product	6	6.9
Industrial chemicals	5	5.7
**Pre-hospital intervention actions taken after poisoning**		
Nothing	47	54.0
Self-care	28	32.2
Call physician	1	1.1
Consult physician	7	8.0
Consult pharmacist	3	3.4
Consult traditional healer	1	1.1

Of all the hospitalised patients, 77 (88.5%) were admitted to the emergency department and 5 (5.7%) were admitted to the ICU. All poisoning cases admitted to the hospital didn’t contact the Poison Control Center of Morocco (MPCC), while 32.2% of parents proceed with self-care of their children, 8% consulted a physician and 1.1% consulted traditional healer.

### Direct medical costs per patient

The different laboratory tests administrated according to the patient state were: complete blood count (CBC), biochemical tests, the c-reactive protein (CRP) test, lactate dehydrogenase (LDH), glutamic-oxaloacetic transaminase (GOT), glutamate-pyruvate transaminase (GPT), prothrombin time (PT), bilirubin, reticulocyte test, and triglyceride test. While, the functional and imaging tests included electrocardiography (ECG), brain CT scan, chest radiography and plain film of the abdomen.

**Fig. 1:**
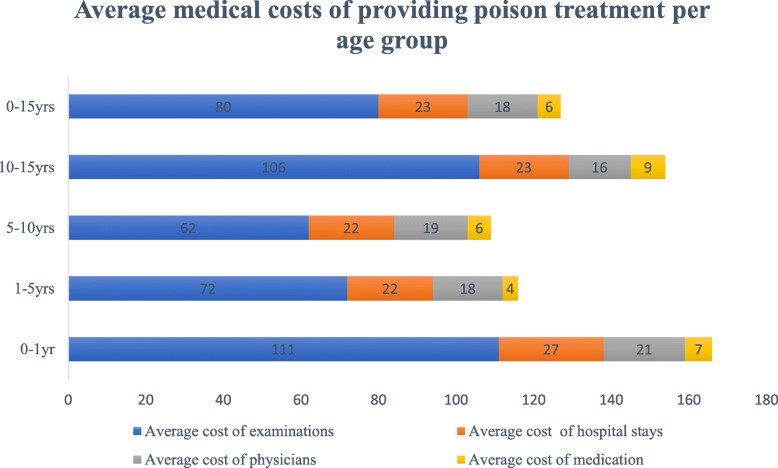
Average medical cost of providing poison treatment per age group at the Children’s University Hospital of Rabat between March and July 2016 (USD, year 2016 values)

Table 2 presents in detail the structure of the direct medical cost. The average direct medical cost of providing poison treatment per child in the Children’s University Hospital of Rabat, Morocco was about USD 127 per child (average cost of examinations USD 80 + average cost of hospital stays USD 23 + average cost of physicians USD 18 + average cost of medication USD 6). The average cost of examinations (laboratory and imaging tests) was significantly higher than the other components of the direct medical costs. The average direct medical cost was significantly higher in children aged less than one year compared to the other children age groups (Fig. 1). while, it was higher for children admitted to the intensive care unit (ICU) than children admitted to the emergency department (ED), with respectively USD 516 per child and USD 110 per child.

**Table 2:** Direct medical costs of poisoning per patient by type of hospital admissions at the Children’s University Hospital of Rabat between March and July 2016 (USD, year 2016 values)

Hospital admissions		Costs of examinations	Costs of hospital stays	Physicians’ costs	Medication costs
ED admissions	Mean^a^	$61 (79)	$24 (20)	$18.66 (10)	$6 (16)
	Min	_	$11	$11	_
	Max	$580	$107	$60	$120
ICU admissions	Mean^a^	$456 (263)	$32 (18)	$24 (7)	$4 (9)
	Min	$304	$21	$18	_
	Max	$912	$64	$36	$21

*SD* Standard Deviation, *Max* Maximum, *Min* Minimum, *ED* Emergency department, *ICU* Intensive Care Unit

^a^Values presented as mean (SD)

### Direct non-medical costs per patient

**Fig. 2:**
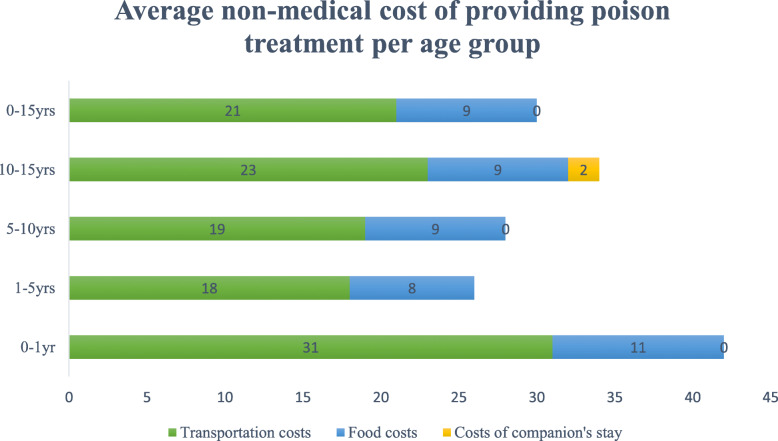
Average non-medical cost of providing poison treatment per age group at the Children’s University Hospital of Rabat between March and July 2016 (USD, year 2016 values)

Table 3 shows the average direct non-medical cost of providing poison treatment for children in the Children’s University Hospital of Rabat, Morocco that was about USD 30 per child. The average direct non-medical cost was higher for children admitted to the intensive care unit (ICU) than children admitted to the emergency department (ED) and non-hospitalised children, with USD 60 per child, USD 29 per child and USD 7 per child, respectively. The total direct non-medical costs were mainly driven by the costs of transportation (Fig. 2) which presented 69% of the total direct non-medical costs. We found that an ambulance and a taxi are the most used means of transport.

**Table 3:** Direct non-medical costs of poisoning per patient by type of hospital admission at the Children’s University Hospital of Rabat between March and July 2016 (USD, year 2016 values)

Hospital admissions		Transportation costs	Food costs	Costs of companion’s stay	Total non-medical costs
ED admissions	Mean^a^	$20 (18)	$9 (7)	_	$29 (22)
	Min	$3	$4	_	$7
	Max	$100	$36	_	$115
ICU admissions	Mean^a^	$39 (35)	$15 (20)	$6 (13)	$60 (65)
	Min	$8	$4	_	$12
	Max	$91	$50	$30	$171
Non-hospital admissions	Mean^a^	$7 (2)	_	_	$7 (2)
	Min	$5	_	_	$5
	Max	$11	_	_	$11

*SD* Standard Deviation, *Max* Maximum, *Min* Minimum, *ED* Emergency department, *ICU* Intensive Care Unit

^a^Values presented as mean (SD)

### The length of stay characteristics

Table 4 presents the length of stay (LOS) for children with poisoning at the Children’s University Hospital of Rabat, the mean ± SD length of stay (LOS) was 2.15 ± 1.87 days with a range variated between 0 day and 10 days of hospital stay.

**Table 4:** Hospital length of stay (LOS) of children at the Children’s University Hospital of Rabat between March and July 2016

	Mean (Median)/SD	(n)
LOS range: 0–10	2.15 (2)/1.87	
**Distribution (days):**		
0		5
1 day		35
2 days		26
3 days		10
4 days		1
5 days		3
6 days		1
7 days		5
10 days		1
**Total**		**87**

Of the admitting children, we found that drugs, snakebites and scorpion stings, plants and petroleum products poisonings were associated with the longest LOS which varied between 6 and 10 days of hospital stays.

## Discussion

To the best of our knowledge, this is the first study in Morocco that evaluated the direct costs of childhood poisoning by estimating the medical and non-medical costs. In Morocco, all the articles published on poisoning analyse and discuss the morbidity impact of patients and neglect to quantify the economic burden of poisoning.

In the results of this study, the average length of stay for children with poisoning diagnoses was 2.15 ± 1.87 days with a range variated between 0 day and 10 days of hospital stay and 24% of all children had a length of stay equal to or longer than 3 days. In an Australian study, Lee and Colleagues reported that the inpatients admitted to a combined general medicine and toxicology services experienced a shorter length of stay than patients not consulted by toxicologists [12]. Different studies show that the mean length of hospital stay for patients with poisoning usually reported being shorter than the current study, one to two days [13–15]. The patients managed via the Poison Control Center experienced shorter LOS and utilised the emergency departments less frequently [16–18] which can explain the high average LOS in the current study where no one of the poisoning cases contacted the Poison Control Center of Morocco (MPCC) before the hospital intervention.

The total burden of poisoning in this research was estimated to USD 13,620. Direct medical costs accounted for 80% against 20% of direct non-medical costs. The average direct medical and non-medical costs of providing poison treatment were respectively USD 127 and USD 30 per child’s (total of USD 157). The average direct cost of poisoning USD 157 represented more than half (60%) of the national minimum wage per month in Morocco, and it is almost the same as the cost reported in Nigeria (USD 168) [2].

From this study, 80% of companions were economically inactive (in the current study, companions of children were mostly mothers). Thus, the average number of work-loss days and the productivity losses associated with the children poison treatment were not estimated. It should be recognized that, in general, the indirect costs of poisoning can merely be equal to the direct costs or more, especially in the case of deaths related to poisoning.

In the international literature, different countries mainly in the developed countries have evaluated the cost of poisoning by estimating the direct and indirect related costs. Whereas, our study refers only to the direct costs of poisoning, the importance of the indirect costs has been emphasized internationally and exceed direct costs worldwide. In the USA, medical spending for poison treatment totalled USD 3 billion in 1992 [19] and 16.4 million of work-loss days. The childhood poisoning caused USD 23.7 billion of productivity loss in 2000, that was increased to more than USD 33 billion in 2010 [20]. In Canada, the direct costs for unintentional poison treatment totalled USD 86 million, and USD 229 million for the indirect costs in 2013. Moreover, the direct costs for Suicide/self-harm due to poisoning totalled USD 143 million and USD 107 million for the indirect costs [7, 21].

### Limitations

The results obtained in this study reflect only the poison treatment during the length of stay from arrival at the hospital until departure and do not include the indirect costs of parents’ absenteeism from work or the costs occurred before that patients were referred to the hospital and after their discharge; for example, in cases of the psychotherapy sessions that the children needed to get after suicide attempts. Therefore, future study that takes into consideration the indirect costs related to childhood poisoning would be beneficial, as the costs explored in this study might have been underestimated because the indirect costs have not been taken into consideration.

## Conclusions

Considering that the cost of poisoning has not been estimated before in Morocco, this study showed that the childhood poisoning associated with significant costs. Yet, the prevalence and the costs related to the unintentional childhood poisoning can be prevented by improving the parents’ awareness, parents’ socioeconomic status and parents’ literacy.

## Data Availability

The datasets used and analysed during the current study are available from the corresponding author on reasonable request.

## Abbreviations

IHME: Institute for Health Metrics and Evaluation
GBD: Global Burden of Disease Study
MPCC: Poison Control Center of Morocco
USD: United States Dollars
ICU: Intensive Care Unit
ED: Emergency department
CBC: Complete Blood Count
CRP: C-Reactive Protein test
LDH: Lactate Dehydrogenase
GOT: Glutamic-Oxaloacetic Transaminase
GPT: Glutamate-Pyruvate Transaminase
PT: Prothrombin Time
ECG: Electrocardiography
Max: Maximum
Min: Minimum
SD: Standard Deviation
